# Evaluation of the serum β2 Microglobulin level in patients with systemic lupus erythematosus and its correlation with disease activity

**DOI:** 10.1051/bmdcn/2019090316

**Published:** 2019-08-27

**Authors:** Miramir Aghdashi, Simak Salami, Ahmad Nezhadisalami

**Affiliations:** 1 Urmia University of Medical Sciences Urmia Iran; 2 Shahid Beheshti University of Medical Sciences Tehran Iran

**Keywords:** β2 microglobulin, Disease activity, SLE

## Abstract

Background: Designation of disease activity is serious for the management of systemic lupus erythematosus (SLE). Serum level of β2 microglobulin (β2M) may be associated with illness activity in SLE disease. Since the role of β2M for assessing of illness activity in SLE is not completely clear, the current study aimed to discern evaluation of β2M in patients with SLE and its correlation with sickness activity.

Materials and Methods: In this case-control study, 50 patients with SLE disease and 25 healthy individuals were selected in Imam Khomeini Hospital in central of Urmia.

Blood samples were collected safely from patients, serum was removed, and β2M measured using an ELISA method. The results for other parameters including C reactive protein, C3, C4, anti dsDNA and erythrocyte sedimentation rate were obtained from patients’ medical record. Data analyzed using appropriate statistical tests including Mann-Whitney U test, Independent f-test, *Kruskal-Wallis,* and Spearman used for analysis of data.

Results: In the current study, a significant difference was seen between two groups in terms of β2M (p < 0.001). Remarkable correlation was seen between the level of β2M with disease activity (p < 0.001). Furthermore, there are significant relevancy between the level of β2M with 24-hour urine protein, ESR, disease activity score, and CRP (p < 0.05).

Conclusion: The results revealed that serum amount of β2M in SLE patients is higher compared to healthy ones, which is significantly correlated to score of illness activity, CRP, and ESR in patients with SLE disease. Hence β2M might be an excellent serological marker helping the prediction of sickness activity and inflammation in SLE patients.

## 1. Introduction

Systemic lupus erythematosus (SLE) is a serologically and clinically heterogeneous illness [1] which leads to malfunction of the immune system [2–13]. It is a multiorgan disease stemmed from the production of a broad span of antinuclear antibodies and existence of immune complexes in the involved organs [14]. Moreover, activation of T and B cells in SLE leads to the generation of autoantibodies and damage of tissues [1], evoking loss of self-tolerance. The tolerance loss following the deregulation of the immune system is due to genetic and environmental factors as well as stochastic events [15–18]. More than 30 genetic loci are involved in the pathogenesis of SLE [15].

According to previous study, the proper assessment of SLE activity and determination of its grade can identify suitable therapeutic regimes and manage the disease [19].

The current tools for estimation of disease activity are serum (anti-ds DNA) anti-double-stranded DNA antibodies, C3 and C4 complement components, and anti-C1q antibody [20–23]. CRP is a non-specific marker of various inflammatory situations, but it may be considered as a direct scale of disease or as a prognostic indicator of autoimmune diseases [4]. Also, β2 microglobulin (β2M) is known as a low weight protein (11 kDa) existing in the all nucleated cell surfaces as section of main histocompatibility complex [1]. Its daily formation is 50-200 mg with a plasma half-life of 2 hours [24]. But it is proved that lymphocyte activity during lymphoproliferative and autoimmune illness can also alter the plasma level of β2M [25, 26]. The reabsorption of circulating β2M occurs in proximal renal tubule [27, 28] and the enhanced urinary β2M is a well-known sign of tubule interstitial renal diseases [25].

High amount of serum β2M have been reported in rheumatoid arthritis disease, Sjogren’s syndrome, and Systemic lupus erythematosus [29–31]. In addition, the level of β2M is as an index of illness activity for evaluating of SLE disease [25]. However, the association of serum amount of β2M with SLE disease activity has not clearly elucidated yet. Since few studies have been done for evaluation of serum β2M level in patients with systemic lupus erythematosus and its correlation with disease activity in our country, the current study aimed to discern serum levels of β2M in SLE patients and its correlation with illness activity, CRP, and other parameters.

## 2. Materials and methods

### 2.1. Designing of study

In current cross-sectional survey, 50 patients with diagnosed SLE disease from Imam Khomeini Hospital in central of Urmia, and 25 healthy individuals were included. All tests were performed in laboratory of Imam Khomeini Hospital. After giving consenting and approving of current study by Ethical Committee of University, demographic, clinical and other data of patients were obtained from medical records.

### 2.2. Criteria of sample selection

In this study, patients with definitive and deterministic diagnosis of SLE were selected, but the cases with concurring diabetes mellitus, endocrine disease, multiple myeloma, blood cancer and concomitant infection were excluded.

### 2.3. Distinguishing of SLE illness activity

The score of SLE illness activity was calculated through the SLE- DAI or Systemic Lupus Erythematosus Disease Activity Index scoring guideline (SLEDAI 2000). Patients with SLE were classified into two subgroups of patients with score of less or more than 8.

### 2.4. Determining of β2M

A blood sample was collected via a safe vein puncture, and serum samples kept frozen at -20°C. β2M was measured using ELISA method (Pars Azmoon Co., Tehran, Iran)with a detection level of 0.1 mg/L. All steps were done according to the instructions.

### 2.5. Determing of CRP

CRP level was regularly evaluated for all patients during the first 24 hours. Nephlometery technique was used for assessment of human (Pars-Azmoon test kit, Tehran, Iran). All steps were done according to the instructions.

### 2.6. Determining of ESR

Erythrocyte sedimentation rate is a common hematologic test for the diagnosis of non-specific inflammation. When anticoagulant blood was transferred into the glass tube and placed immoble, the red blood cells dissipate from the plasma and precipitate. The speed at which red blood cells precipitate is measured as a transparent plasma millimeter appearing above the tube after an hour.

### 2.7. Determing of C3 and C4

A Kinetic Nephelometric technique was used for assessment of Serum C3 and C4 using commercial kit (Pars Azmoon, Tehran, Iran). All steps were done according to the instructions.

### 2.8. Determining of anti-dsDNA antibodies

The Farr assay (^125^I-labelled recombinant dsDNA) was used to determine serum anti-dsDNA antibodies according to manufacturer’s instruction (UK).

## 3. Statistical analysis

In current study, data were entered SPSS version 20. For comparison of case and control in terms of β2M and age, we used MannWhitney U test and Independent t test, respectively. For comparison of patients with respect to disease activity, Kruskal-Wallis was used. Spearman was applied for correlation of β2M with clinical data. *P* < 0.05 was considered for statistical analysis.

## 4. Results

The mean value of clinical parameters of patients with SLE are summarized in Table 1.

**Table 1:** The mean value of clinical data (ESR, Anti-ds DNA, C3, C4, Score of disease, duration of disease, age and 24 hour urine protein) in patients with SLE

Variable	Mean ± SD	Minimum	Maximum
ESR (mm/h)	37.76 ± 4.73	5	110
Anti-ds DNA	2.48 ± 0.23	0.2	6.70
C3	1.02 ± 0.07	0.07	1.70
C4	0.3 ± 0.04	0.05	1.90
Score of disease	8.04 ± 1.01	0	27
Duration of disease (day)	41 ± 6.72	1	204
Age (year)	32.01 ± 1.61	16	62
24-hour urine protein (mg)	1185 ± 242.01	0	6150

ESR: Erythrocyte Sedimentation Rate

Anti-ds DNA: Anti-double stranded DNA

C3: Complement component 3

C4: Complement component 4

No significant difference was organized between age of case (32.01 ± 1.61) and control (30.28 ± 1.11) groups (*p* = 0.37).

Table 2 presentes the status of CRP in patients with SLE.

**Table 2:** - Frequency distribution of CRP in patients with SLE.

CRP level	Frequency	Percent (%)
0	26	52
+ 1	16	32
+2	5	10
+3	3	6

0; Normal, 1: Mild inflammation, 2: Moderate inflammation, 3: Sever inflammation

As clear from table 2, only 6% of patients with SLE had CRP score +3.

Table 3 shows comparison of serum levels of β2M in patients with SLE and control.

**Table 3:** Comparison of serum levels of β2M in patients with SLE and control group.

Variable	Case	Control	*p*-value
			Mann-Whitney U test
Serum β2M- (ng/*dl*)	6.52 ± 0.77	2.80 ± 0.26	0.001

As shown in table 3, a significant difference was seen in serum levels of β2M in SLE patients with healthy subjects (p < 0.001).

Table 4 summarized β2M level in SLE patients and subjects of control.

**Table 4:** The comparison of serum amount of β2 microglobulin in SLE patients and healthy subject.

The level of β2 microglobulin (ng/dl)	Mean ± SD	Confidence interval	Min	Max	*p*-value
					Kruskal-W allis
Active	7.97 ± 1.21	5.48-10.47	1.35	19.95	0.001
Inactive	5.08 ± 0.88	3.26-6.90	1.39	19.95	
Control	2.80 ± 0.26	2.25-3.33	1.20	5.96	

As domonestrated in Table 4, remarkable difference in serum levels of β2M was found between healthy subjects and patients with active and inactive SLE (p < 0.001).

Table 5 showed the correlation of β2M with duration of disease, SLEDAI, ESR level, CRP, anti-ds DNA, C4, C3 and 24 urine protein in SLE patients.

**Table 5:** The correlation of β2 Mwith duration of disease, SLEDAI, ESR level, CRP, anti-ds DNA, C4, C3 and 24 urine protein in SLE patients.

Variables	The level of β2Microglobuline (ng/*dl*)	p-value
	Spearman correlation coefficient	
Duration of disease (day)	-0.26	.07
Score of disease activity (SLEDAI)	0.39	0.001
ESR level (mm/h)	0.33	0.02
CRP level	0.34	0.01
Anti- ds DNA (IU/ml)	0.15	0.31
C4 components	-0.02	0.90
C3 components	-0.13	0.39
24-hour urine protein (mg/24 h)	0.37	0.01

SLEDAI: systemic lupus erythematosus Disease Activity Index

ESR: Erythrocyte Sedimentation Rate

CRP: C reactive protein

Anti- ds DNA: Anti-ds DNA: Anti-double stranded DNA

C3: Complement component 3

C4: Complement component 4

Significant relevancy was seen between the serum amount of β2M with age, a score of disease activity, ESR, CRP and 24-hour urine protein (*p* < 0.05) (Table 5). However, no significant correlation was observed with C3 and C4, duration of disease, anti dsDNA (*p* > 0.05).

**Fig. 1:**
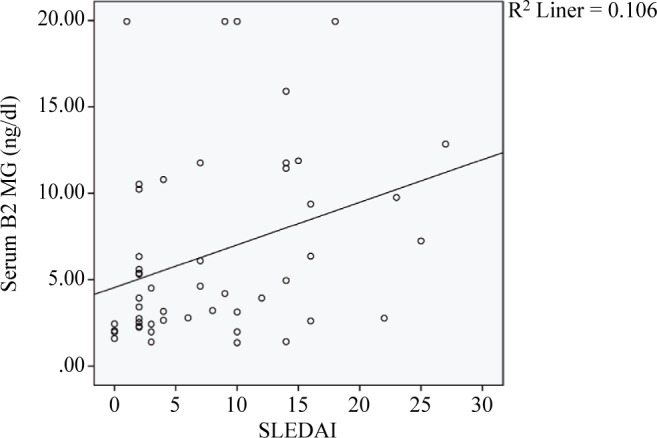
Correlation of Serum Β2M with score activity disease in SLE patients.

Fig. 1 shows correlation between serum β2M with score activity disease in patients with SLE.

Spearman coefficient correlation was used for correlation of serum β2M with score activity disease in patients with SLE and reported 0.39.

## 5. Discussion

SLE as an autoimmune disease is associated with many immunological changes such as T cell abnormality and B cell hyperactivity. Recently, the significance of β2M for assessing of SLE activity has been highlighted.

In current study, significant relevancy was seen between case and control groups in terms of β2M, so that lower level of β2M was detected in healthy control compared to SLE patients. Kim *et al.,* in similar study evaluated the level of β2M through ELISA in normal healthy one and patients with SLE disease [2] and reported that theses patients have higher level of β2M than healthy control. Yeung *et al.,* measured the amount of serum β2M in 115 patients with SLE [32] and observed increased level of serum β2M in 16.4% of patients with external manifestation of SLE [32]. Iwona *et al.,* conducted a study on 100 SLE patients and observed increased level of β2M in 97% of them [33]. Hermansen *et al.,* also demonstrated that the concentration of β2M was remarkably higher in SLE patients [34]. The reason of elevated amount of β2M in SLE disease has not been completely clear. Such an increase might be due to turnover of lymphocytes during lympho proliferative and autoimmune illness [35–38] or presence of immune complexes (β2M and anti-β2M antibodies) which is elemi- nated by kidney [33]. But, Wakabayashi *et al.,* showed that β2M level was decreased during the immunosuppressive treatment [39].

Furthermore, we observed remarkable correlation between disease activity and serum level of β2M in SLE patients Moreover, Choe Y *et al.,* achieved similar result and reported that the level of β2M is accompanied with disease activity in women with SLE [25]. Kim *et al*., showed that the level of serum β2M can be as an excellent biomarker for determining of disease activity in patients with SLE [2]. Lwona *et al.,* reported that increased level of β2M is correlated with disease activity including SLEDAI [33]. Hermansen *et al*., demonstrated significant correlation between SLEDAI score and β2M [34]. Choe *et al.,* evaluated urine β2M and SLEDAI in patients with SLE and reported that there was significant association between urine SLEDAI and β2M [25].

In another study, the level of β2M was assumed for distinguishing of disease activity [32]. According to findings of aforementioned studies, it seems that β2M can be assumed as a good parameter for detecting SLE activity [33].

In current study, remarkable relevancy was observed between serum β2M and 24 hour urine protein. Choe *et al*., also observed a relevancy between renal involvement and disease activity in SLE didease [25]. Badr *et al,* reported that β2M is an accurate and sensitive marker for evaluating of renal action. They also reported that there is significant relation between β2M and glomerular filtration rate (GFR) [4].

Moreover, we observed a significant relevancy between serum level of β2M with CRP and ESR. Skare *et al*., evaluated the correlation between serum level of β2M and ESR. They found a positive correlation between SLEDAI and ESR which is consistent with our study [19]. Rezaeiyazdi *et al.,* observed higher level of CRP in patients with lupus disease compared to healthy control. But they reported that this marker could not be as a good indicator for disease activity [39]. Enocsson *et al*., evaluated serum level of CRP in 155 SLE patients and 100 controls. They saw a powerful relevancy between the level of CRP and disease activity. They also reported that CRP genotype affects the response of CRP in patients with SLE. Therefore, it seems that polymorphisms in the CRP gene can influence CRP answer in these patients [40].

Furthermore, no significant relevancy was seen between serum level of β2M with anti-dsDNA antibodies and C3, as well as C4in current study. However, Żychowska *et al.,* observed the correlation between the level of β2M with complement of C4, C3 and anti-dsDNA [23] which is inconsistent with our study. It seems that one of the possible reasons is due to methods used for measurement of these biomarkers. In Żychowska’ s study, C4 components were measured by turbidimetry method, and antidsDNA antibody was evaluated by ELISA technique, while in our study, the ^125^I Farr assay and nephelometric technique were used for assessment of anti-dsDNA and C4, respectively. Moreover, different kits used in various studies is other influential factor on results and findings. In addition, sample size of Żychowska’s study was greater than our study.

Skare *et al.* evaluated the level of β2M and C3 in systematic lupus disease and reported a inverse relevancy between the level of β2M and C3 [19]. We also observed inverse relevancy between β2M and C3, but this correlation was not statistically emi- ment which could be due to our smaller sample size than Skare *et al.,* study. In addion, current study did not achive significant correlation between C4 component and β2M, while Lewona *et al.,* observed significant association between C4 component and β2M [33]. Maybe it is due to small sample size. We assessed this study on 50 patients, wherease, Lewona *et al.*, performed this study on larger sample size.

## 6. Conclusion

According to data of current study, serum level of β2M is increased in SLE patients which is correlated to score of activity of disease, CRP and ESR. It seems that β2M may be a favorable biomarker and excellent serological marker in laboratory tests to assess disease activity of SLE.

## 6. Conflicts of interest statement

The authors wish to disclose no conflicts of interest.
